# Diagnosis of penile fracture in primary care: a case report

**DOI:** 10.4076/1757-1626-2-8065

**Published:** 2009-07-30

**Authors:** Sevgi Ozcan, Ersin Akpinar

**Affiliations:** Department of Family Medicine, Cukurova University Faculty of MedicineBalcali 01330, AdanaTurkey

## Abstract

**Introduction:**

Penile fracture has been reported with sexual intercourse, masturbation, rolling over or falling on to the erect penis. Classically the history is with a sudden snap, pain, detumescence and a hematoma of the penis with deformity. Immediate surgical treatment is recommended. The patients may delay the admission due to fear and embarrassment or the condition may usually be underreported.

**Case presentation:**

A 32-year-old man presented to primary care complaining of discoloration of penis without any significant history or symptom. Physical examination revealed swollen, ecchymotic, and deviated circumcised penis.

**Conclusion:**

Although frequent and common diseases represent the majority of daily work, the primary care physician should be alert for possible unexpected history or symptom of a rare and often serious condition.

## Introduction

Traumatic injuries to the genitalia are not common in men, because of the mobility of the penis and scrotum [1]. Penile fracture, or penile rupture, is the most common blunt injury to the erect penis and is caused by tearing or cracking of the corporal cavernosal bodies [2]. It can be accompanied by urethral rupture or by injury of the dorsal nerve and vessels. Although it has been reported most commonly with sexual intercourse, it has also been described with masturbation, rolling over or falling on to the erect penis [1-3].

The first documented report of this injury was more than 1000 years ago. More than 1600 cases of penile fracture appeared in the medical literature from 1935 to 2001, 56% of them reported from Mediterranean countries. The largest series were from the United States and Canada (250), Iran (240), Morocco (226), and Turkey (114) [1]. It is usually underreported, because of reluctance and shame that victims experience to seek treatment [1-4].

## Case presentation

**Figure 1.**
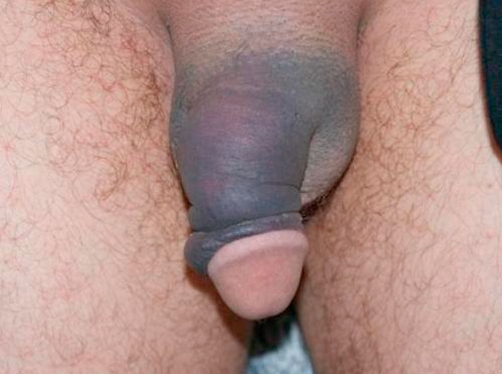
Swollen, ecchymotic, and deviated circumcised penis.

A 32-years-old Turkish man presented to primary care complaining of discoloration of penis since the night before. He did not give any significant sexual or trauma history. Systemic investigation revealed no significant symptoms including pain. His height was 1.58 m, body weight 55 kg, body mass index 22, and blood pressure 115/80 mmHg. Physical examination revealed swollen, ecchymotic, and deviated circumcised penis (Figure 1).

Although he did not give any history of trauma, penile fracture was suspected and it was thought that he was shamed. *“It may be penile fracture. It has been described with sexual intercourse, masturbation, rolling over or falling on to the erect penis. Patient usually describes a cracking or popping sound, followed by pain, and discoloration and swelling of penile shaft. If you had a similar history, you should undergo immediate surgical treatment.”* After this explanation the patient said *“Yes, I had”,* with fear and embarrassment, but he did not want to tell the details. He was referred to the urologist for investigation and treatment. He was hospitalized. Magnetic Resonance Imaging (MRI) revealed “penile fracture”.

## Discussion

Penile fracture may present with classic “eggplant deformity” of swollen penis along with ecchymosis confined to Buck’s fascia [2]. The patient age ranges from 12 to 82 years with a mean age of mostly fourth decade [3]. The patient usually describe a cracking or popping sound as tunica tears, followed by pain, rapid detumescence, and discoloration and swelling of penile shaft [1-3].

Fracture typically occurs during vigorous sexual intercourse, when the rigid penis slips out of the vagina and strikes the perineum or pubic bone, sustaining a buckling injury. Although penile fracture has been reported most commonly with sexual intercourse it can happen from any type of blunt trauma affecting the tumescent shaft. This includes masturbation, with or without devices; falling out of bed with an erection; extreme sexual activity, especially during coitus in which the female is on top; forceful correction of a congenital chordee; and even tucking an erect penis into underwear. In the Middle East, self-inflicted fractures predominate [1-3].

Penile rupture can usually be diagnosed based solely on history and physical examination findings; however, in equivocal cases, diagnostic cavernosography or MRI should be performed [1-3]. Because fear and embarrassment are commonly associated; the patient’s presentation to the health care professionals is sometimes significantly delayed. In our case, first the patient did not give any significant history probably due to embarrassment. After our explanation of the disease and necessity of immediate surgical treatment he accepted the history but he did not want to tell the details.

False fracture has been reported in patients who present with penile swelling and ecchymosis, although they do not describe classic “snap-pop” or rapid detumescence typically associated with fracture. Physical examination may not be adequate for definitive diagnosis in these cases. Another condition that may mimic penile fracture is rupture of the dorsal penile artery or vein during sexual intercourse [2].

Treatment may be either conservative or surgical. The conservative management of penile fracture includes splinting, cold compresses, and a combination of anti-inflammatory, analgesic medications and fibrinolytics. However, long term outcomes of conservative management demonstrated significant complication rates, such as curved or painful erections, erectile dysfunction, arteriovenous fistula formation, infection and plaque formation [1-3]. Suspected penile fractures should be promptly explored and surgically repaired. Immediate surgical reconstruction results in faster recovery, decreased morbidity, lower complication rates, and lower incidence of long term penile curvature [3]. In our case, because of the delay, the patient and the urologist preferred conservative management.

Although it was reported that the fourth largest series in literature were from Turkey, this is the first case we had in our 17 years’ clinical experience in primary care. There are several case reports or series from urology or emergency but not one from primary care. It is unclear whether patients do not admit to primary care or primary care physicians missed diagnose or do not report penile fracture. Mallen mentioned that “As general practitioners, we share cases with our clinical partners everyday and we learn from these informal experiences but we have no real tradition of publishing case reports” [5].

## Conclusion

We present a 32 years-old man admitted for discoloration of the penis and diagnosed as penile fracture in a primary care setting. The aim of this presentation is to share our experience about this rare and underreported condition and to emphasize the importance of immediate surgical treatment. Although frequent and common diseases represent the majority of daily work, the primary care physician should be alert for possible unexpected history or symptom of a rare and often serious condition. Furthermore, detailed medical history including patient’s fears and worries, patient education and shared decision making will help patient to understand the doctor’s uncertainty.

## Abbreviation

MRI: Magnetic Resonance Imaging
